# An Efficient YOLO Algorithm with an Attention Mechanism for Vision-Based Defect Inspection Deployed on FPGA

**DOI:** 10.3390/mi13071058

**Published:** 2022-06-30

**Authors:** Longzhen Yu, Jianhua Zhu, Qian Zhao, Zhixian Wang

**Affiliations:** 1 College of Economics and Management, Qingdao University of Science and Technology, Qingdao 266000, China; yulongzhen@qust.edu.cn (L.Y.); wangzhixian62@126.com (Z.W.); 2 Department of Creative Informatics, Kyushu Institute of Technology, Fukuoka 804-8550, Japan; cho@ai.kyutech.ac.jp

**Keywords:** vision, defect inspection, YOLO, FPGA, attention

## Abstract

Industry 4.0 features intelligent manufacturing. Among them, the vision-based defect inspection algorithm is remarkable for quality control in parts manufacturing. With the help of AI and machine learning, auto-adaptive instead of manual operation is achievable in this field, and much progress has been made in recent years. In this study, considering the demand of inspection features in industrialization, we made further improvement in smart defect inspection. An efficient algorithm using Field Programmable Gate Array (FPGA)-accelerated You Only Look Once (YOLO) v3 based on an attention mechanism is proposed. First, because of the relatively fixed camera angle and defect features, an attention mechanism based on the concept of directing the focus of defect inspection is proposed. The attention mechanism consists of three improvements: (a) image preprocessing, which is to tailor images for selectively concentrating on the defect relevant things. Image preprocessing mainly includes cutting, zooming and splicing, named CZS operations. (b) Tailoring the YOLOv3 backbone network, which is to ignore invalid inspection regions in deep neural networks and optimize the network structure. (c) Data augmentation. First, two improvements can be made to efficiently reduce deep learning operations and accelerate the inspection speed, but the preprocessed images are similar and the lack of diversity will reduce network accuracy. So, (c) is added to mitigate the lack of considerable amounts of training data. Second, the algorithm is deployed on a PYNQ-Z2 FPGA board to meet the industrialization production requirements for accuracy, efficiency and extensibility. FPGA can provide a low-latency, low-cost, high-power-efficiency and flexible architecture that enables deep learning acceleration for industrial scenarios. A Xilinx Deep Neural Network Development Kit (DNNDK) converted the improved YOLOv3 to Programmable Logic (PL), which can be deployed on FPGA. The conversion process mainly consists of pruning, quantization and compilation. Experimental results showed that the algorithm had high efficiency, inspection accuracy reached 99.2%, processing speed reached 1.54 Frames per Second (FPS), and power consumption was only 10 W.

## 1. Introduction

Manufacturing involves a large number of parts. However, installation, welding, handling and many other sectors of manufacturing inevitably cause part defects, most of which can be identified by vision. The vision-based defect inspection algorithm is crucial to ensure the quality of parts and the entire manufacturing process. Industry 4.0 features intelligent manufacturing, which means doing jobs as efficiently as possible and adapting quickly to new conditions. With the help of AI and machine learning, auto-adaptive operation is replacing manual operation in defect inspection. In particular with the emergence of cutting-edge deep learning technologies [1], the scope intelligence, accuracy, speed and efficiency of defect inspection algorithms are improved significantly [2].

Deep learning algorithms for vision-based defect inspection can be mainly divided into two types: classification-based algorithms and regression-based algorithms [3]. Algorithms based on classification are represented by Region-based Convolutional Neural Network (R-CNN) series, including R-CNN, Spatial Pyramid Pooling Networks (SPP-Net), Fast R-CNN, Region-based Fully Convolutional Networks (R-FCN), and Mask R-CNN. Based on these algorithms, Fan et al. [4], Ji et al. [5], Zhang et al. [6], Guo et al. [7], Jin et al. [8] and Cai et al. [9] have inspected surface defects of wood, gear, metal, etc. Using two-stage processing (region extraction and object classification), R-CNN algorithms generally require high computing power to achieve high accuracy but with a relatively low inspection speed. Regression-based algorithms are characterized by only one round of processing, so the speed is faster. Redmon et al. [10] proposed the well-known You Only Look Once (YOLO) algorithm, which is a representative regression-based and end-to-end model. To date, the YOLO series has evolved to include YOLOv1, YOLOv2, YOLOv3 [11], YOLOv4 [12] and YOLOv5 [13]. Furthermore, the representative regression-based algorithms also include Single Shot MultiBox Detector (SSD) [14] and CornerNet [15]. YOLOv3 is among the most widely used YOLO algorithms. Based on YOLOv3, Jing et al. [16], Li et al. [17], Huang et al. [18] and Du et al. [19] performed surface defect inspection of fabric, PCB boards, pavements, etc.

**Figure 1:**
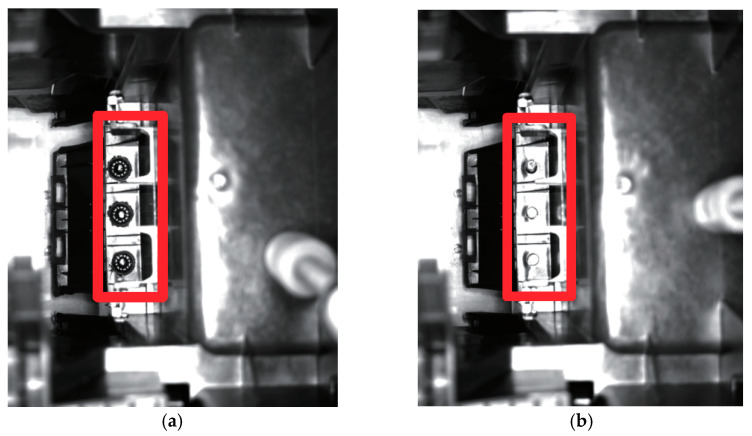
Normal welding and defect welding. (**a**) Normal welding. (**b**) Defect welding.

Compared with classical deep learning object detection algorithms, vision-based defect inspection can be optimized due to two characteristics: first, the recognition region on an image is predictable. As shown in Figure 1, the camera angle of the two parts is fixed. In fact, we only care about the red box region of the two photos. By identifying only this region, we can identify whether the part is defective. Other regions of the original photo can be deleted accordingly. Second, the algorithm needs to meet the deployment requirements of industrial scenarios. The indicators of efficiency must be considered, such as stability, scalability, higher speed and lower power consumption. The target system requirements for this work are as follows. Inspection accuracy should be higher than 97%, image processing speed should be higher than 1 FPS, and the power consumption of each equipment should be less than 100 W.

In this work, an efficient YOLO algorithm for vision-based defect inspection with an attention mechanism deployed on FPGA is proposed. There are two main contributions.

- The camera angles of industry cameras and defect features are relatively fixed. So, an attention mechanism is proposed that is based on the concept of drawing global dependencies between the input and the output of a neural network [20], consequently directing focus on defect inspection. The improvement in attention focuses on three aspects: (1) we use image preprocessing named CZS operations for recombining the defect regions of an image and deleting useless regions. (2) We tailor the YOLOv3 backbone network, remove unnecessary recognition accuracy, and thus increase processing speed. (3) Finally, data augmentation technology is also used to further improve the accuracy of attention.
- In order to meet the requirements of industrial scenarios, the algorithm is deployed on FPGA. Deep learning generally consumes a lot of computing power, followed by high power consumption. With FPGA, we can customize hardware for accelerating large-scale computing and make the application scalable. A Xilinx PYNQ-Z2 FPGA board is used for deployment of the optimized YOLOv3. With the help of DNNDK [20], the deep learning processor unit (DPU) [21] can easily deploy the case deep learning algorithm, and involves three main steps: pruning, quantization and compilation. Case experiments showed that this results in low latency, low power consumption, extensibility, and efficiency.

## 2. YOLOv3 Based on an Attention Mechanism

### 2.1. Image CZS Preprocessing

**Figure 2:**
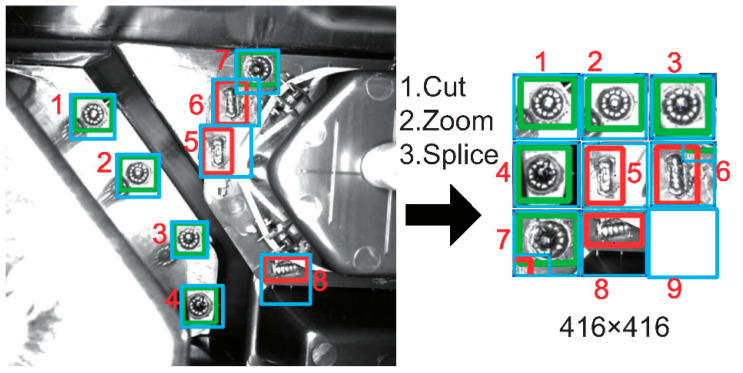
CZS operations.

An attention mechanism focuses on modeling the relationship between the input and the output of the algorithm, regardless of distance [22]. For defect inspection, the camera angles of industrial cameras are relatively fixed. So, we can predefine the possible defect regions and extract features only from the specific region. The self-attention mechanism calculates the sequence semantic representation by associating different positions in the sequence. We add the image preprocessing equivalent to adding a self-attention mechanism to YOLOv3 preprocessing. CZS operations are as shown in Figure 2. The blue box represents the cutting region, and the green box and the red box represent two kinds of defect markup regions. Color boxes numbered 1 to 8 in the original image on the left side correspond to the splicing regions of the image on the right side. All color boxes are the main areas of concern for defect detection. The entire process involved three steps: cut predefined regions from the original image, zoom regions to the same size and splice regions together to form a new image—named CZS operations for short.

Cutting operations take a small square box containing a defect region as a cutting region. The box is slightly larger than the smallest box containing the defect region, so as to ensure fault-tolerant positioning of the same type of images. Define the width of the original image $w_{s}$ and the height $h_{s}$, respectively. The ratio of the width of the center point of the defect region to the original image is $r_{w}$, and thus the width of the center point of the defect region is $w_{m}=w_{s}\times r_{w}$. Similarly, define the height, *x*-coordinate and *y*-coordinate as $r_{h}$, $r_{x}$ and $r_{y}$. Additionally, the height, *x*-coordinate and *y*-coordinate of the defect region’s center point can be expressed as $h_{m}=h_{s}\times r_{h}$, $x_{m}=w_{s}\times r_{x}$ and $y_{m}=h_{s}\times r_{y}$. Define the width, height, *x*-coordinate and *y*-coordinate of the cutting box’s top left corner as $w_{c}$, $h_{c}$, $x_{c}$ and $y_{c}$, respectively.

(1) $$w_{c}=h_{c}=max(w_{m},h_{m})\times \alpha$$

(2) $$x_{c}=\left\{ \begin{array}{l} x_{m}-\frac{w_{c}}{2},x_{m}+\frac{w_{c}}{2}\leq w_{s}\\ w_{s}-w_{c},x_{m}+\frac{w_{c}}{2}>w_{s} \end{array}\right.$$

(3) $$y_{c}=\left\{ \begin{array}{l} y_{m}-\frac{h_{c}}{2},y_{m}+\frac{h_{c}}{2}\leq h_{s}\\ h_{s}-h_{c},y_{m}+\frac{h_{c}}{2}>h_{s} \end{array}\right.$$

The *α* is the expansion coefficient, which takes a value between 1 and 2. This means that cutting box is 1- to 2-fold larger than the smallest box containing the defect region. Formulas (2) and (3) mean that when the defect region is close to the boundary of the original image, the top left corner coordinates of the cutting box should be consistent with the original image.

The zooming operation is to scale all cutting boxes on an image to the same size, so that can be fully held by a new 416 × 416 image (the standard image size processed by YOLOv3 is 416 × 416 pixel). According to YOLOv3, images will be scaled to 416 pixels in width (*W*) and height (*H*) before being processed. Suppose the number of cutting boxes on an image is $N_{c}$. So, the number of boxes that can be held in a row ($N_{h}$) or a column ($N_{v}$) of a new image is calculated as Formula (4). The target size that a cutting box should be scaled to is calculated as Formula (5). The scaling factor *β* is calculated as Formula (6). The sqrt function is used to obtain the square root of the passed argument. The ceiling function is used to obtain the smallest integer larger than the passed argument. The floor function is used to obtain the largest integer smaller than the passed argument.

(4) $$N_{h}=N_{v}=ceiling(sqrt(N_{c}))$$

(5) $$w_{z}=h_{z}=floor(\frac{W}{N_{h}})=floor(\frac{H}{N_{v}})$$

(6) $$\beta =\frac{w_{z}}{w_{c}}$$

The splicing operation is to combine cutting boxes from the original image into a new image after being scaled. Splicing mainly consists of two processing works; one is to map a cutting box to the splicing region; another is to map the actual defect markup region to the splicing region. For the first work, we first sort the cutting boxes from small to large according to the $x_{c}$ value. If the $x_{c}$ values of two cutting boxes are the same, then we sort them from small to large according to their $y_{c}$ value. Suppose that a cutting box is ranked as $N^{i}$, $i=0,\ldots ,N_{h}$, then the width, height, *x*-coordinate and *y*-coordinate of the box’s top left corner in the new image are as defined as $w_{\chi }^{i}$, $h_{\chi }^{i}$, $x_{\chi }^{i}$ and $y_{\chi }^{i}$. Operator // represents the round function and % represents the remainder function.

(7) $$n_{h}^{i}=\left\{ \begin{array}{l} N^{i}//N_{v}+1,N^{i}\%N_{v}\neq 0\\ N^{i}//N_{v},N^{i}\%N_{v}=0 \end{array}\right.$$

(8) $$n_{v}^{i}=\left\{ \begin{array}{l} N^{i}\%N_{v},N^{i}\%N_{v}\neq 0\\ N_{v},N^{i}\%N_{v}=0 \end{array}\right.$$

(9) $$w_{\chi }^{i}=h_{\chi }^{i}=w_{z}$$

(10) $$x_{\chi }^{i}=(n_{v}^{i}-1)\times w_{z}$$

(11) $$y_{\chi }^{i}=(n_{h}^{i}-1)\times h_{z}$$

For the second work, the size ratio of the width, height, *x*-coordinate and *y*-coordinate of the center point of an actual defect markup region to the new image is $R_{w}^{i}$, $R_{h}^{i}$, $R_{x}^{i}$ and $R_{y}^{i}$.

(12) $$R_{w}^{i}=\frac{w_{m}\times \beta }{W}$$

(13) $$R_{h}^{i}=\frac{h_{m}\times \beta }{W}$$

(14) $$R_{x}^{i}=\frac{(n_{v}^{i}-1)\times w_{z}+(x_{m}-x_{c})\times \beta }{W}$$

(15) $$R_{y}^{i}=\frac{(n_{h}^{i}-1)\times h_{z}+(y_{m}-y_{c})\times \beta }{H}$$

In addition to the above algorithms, the new image generated may have some blank regions. We filled them with 0 or 255 values, shown as the square labelled 9 on the right-side image of Figure 2. Then, CZS preprocessing of the image is finished.

### 2.2. Tailoring the Backbone Network

According to the size of the inspection target, the YOLOv3 backbone network can be tailored to detect the defect regions more efficiently. 

**Figure 3:**
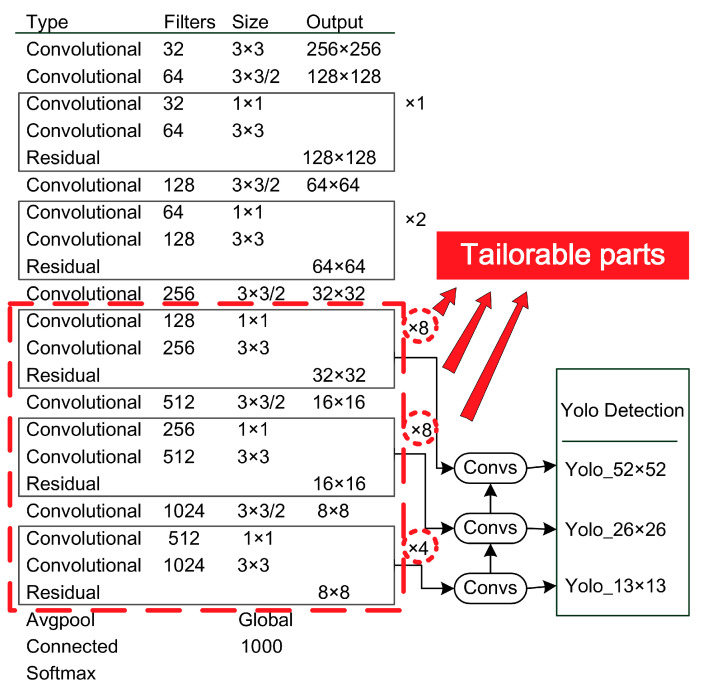
YOLOv3 backbone network and tailorable parts.

As shown in Figure 3, the backbone network of classical YOLOv3 includes 53 layers, so called Darknet-53. Among them, *Convolutional* is the convolution layer, *Residual* is the hop connection layer of residual network, *Avgpool* is pooling layer by average, and *Connected* is the full connection layer. The labels ×1, ×2, ×8, ×8 and ×4 represent repeated execution 1, 2, 8, 8 and 4 times, respectively. Note that the five repeated steps correspond to five down-sampling. Additionally, the outputs of the ×8, ×8 and ×4 down-sampling of the last three steps correspond to the classification prediction feature map (YOLO layer) at three scale resolution levels—52 × 52, 26 × 26 and 13 × 13. The final feature maps of classical YOLOv3 have three sizes, the 52 × 52 resolution has better support for detecting tiny objects, and the 13 × 13 resolution is more suitable for identifying larger objects.

Classical YOLOv3 is used for general object detection, including both large and small objects. The distance of the camera from the object of which photos are taken will also affect the size of the object to be recognized. However, the vision-based defect inspection algorithm is different from classical YOLOv3. The camera angle of industry cameras is relatively fixed, and the shape and size of the defect to be inspected are also relatively fixed. Therefore, based on the fixed shape and size of the defect, only the corresponding resolution networks need to be retained, instead of retaining all three scales (52 × 52, 26 × 26 and 13 × 13) of networks. For example, in the production site of automobile rubber and plastic parts, the visible defect commonly has a moderate size and is obviously distinguished, so the inspection network of such defects does not require a very high resolution. However, on the other hand, in the field of silicon chip solder joint quality inspection, defect inspection of welding points needs high precision. The solder joint layout on the chip is very fine and tiny, so it needs a very high-resolution network for identification. In short, the network structure can be optimized according to targeted inspection tasks. Tailoring the YOLOv3 backbone network can be based on the following formulas.

(16) $$if(\underset{i\in N}{every}(w_{i}>\frac{W}{26}\cap h_{i}>\frac{H}{26})),tailor(yolo_{52\times 52})$$

(17) $$if(\underset{i\in N}{every}(w_{i}<\frac{W}{26}\cap h_{i}<\frac{H}{26})),tailor(yolo_{13\times 13})$$

The *if (condition)* statement is the basic conditional control structure. This allows the *tailor* function to happen, depending on whether a given condition is true. The *tailor* function is used to delete part of the input argument network used to identify a certain scale. The *yolo*_52×52_ is the smallest resolution inspection network part of YOLOv3, while the *yolo*_13×13_ represents the largest resolution network part. The *every* function means every inspected target. The *N* represents the full amount of the inspected targets on an image. The *w*_*i*_ and *h_i_* represent the width and height of a target. The *W* and *H* represent an image’s width and height.

In the case study of chapter 4, the 52 × 52 tiny resolution-scale network is shrunk. That is to partially delete the third down-sampling layers of the backbone network. For classical YOLOv3, the third down-sampling layers consist of eight rounds of repetition. In our algorithm, seven rounds of repetition are tailored off, that is to delete 14 convolutional layers for all. Thus, the backbone network is condensed from 53 layers to 39 layers. Our algorithm’s backbone network turns into Darknet-39.

### 2.3. Data Augmentation

**Figure 4:**
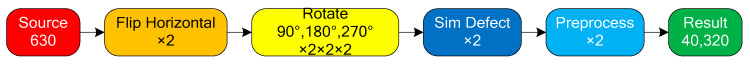
The process of data augmentation.

In order to enhance attention and improve the recognition accuracy of deep learning networks, it is also necessary to implement data augmentation to expand the dataset. The whole process of data augmentation is shown in Figure 4. There are mainly two strategies.

**Figure 5:**
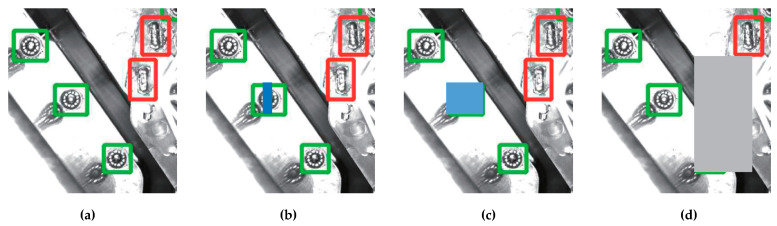
Simulation of noisy or missing faults. (**a**) The normal and original picture marked up with 5 inspection regions of 2 types, green box represents circular solder joint and red box represents strip solder joint. (**b**) One inspection regions was covered by a blue rectangle for 1/3. (**c**) One regions was completely covered by a blue rectangle. (**d**) Two neighbor regions was completely covered by a grey rectangle.

Strategy 1: It is to add random noise to the defect markup region of the original image, which changes from normal to noisy or faulty. As shown in Figure 5, the rectangular cover is used to simulate the noise or missing faults on the surface of the markup region. The position, size and color can be set randomly. From left to right, Figure 5a is normal; Figure 5b uses a rectangle to cover 1/3 region of a markup region, which is equivalent to adding some noise, so it should be ensured that the network training can recognize such markup region; Figure 5c,d completely cover one or two markup regions with rectangles to simulate the missing faults.

**Figure 6:**
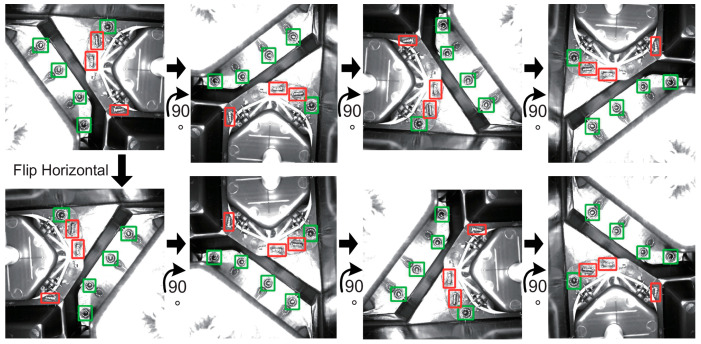
Rotate and flip horizontal the original image.

Strategy 2: It is to rotate the image by 90°, 180° and 270° and flip it horizontally. That is equaled to expand into 8 images by rotation and flipping, as shown in Figure 6. In the original picture, there are a total of 8 regions to be inspected, which are divided into two types, represented by red boxes and green boxes respectively.

The use of a dataset enhancement strategy is not only to improve the quality of training, but also to effectively reduce manpower consumption. In this study, there are only 630 original photos, which can only be manually added to the defect markup region. Then, a python script can be used to automatically complete image preprocessing and dataset enhancement, so that the final dataset size for training and testing reaches 40,320 photos.

## 3. FPGA Deployment

### 3.1. Overall Framework

**Figure 7:**
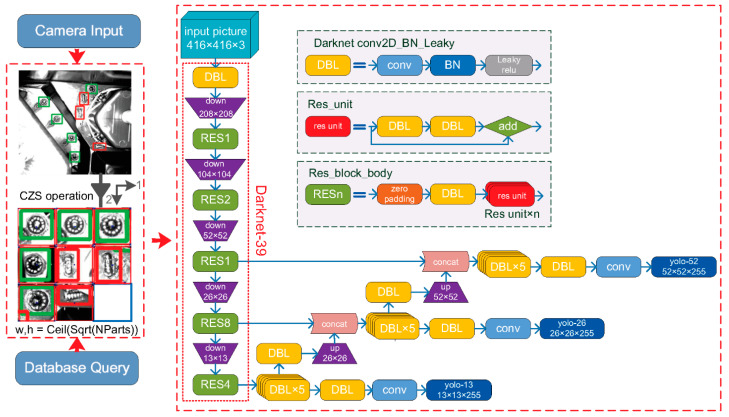
Overall framework of the proposed defect inspection algorithm.

The vision-based defect inspection algorithm is deployed on an All Programmable System on Chip (APSoC), the Xilinx PYNQ-Z2. With its help, we can use low-power-consumption, cutting-edge, customized hardware to replace high-energy-consumption, large-footprint, non-specific-purpose and high-cost deep learning workstations. As shown in Figure 7, the whole algorithm deployed on FPGA includes two parts: image CZS preprocessing, and hardware acceleration for deep learning. Through CZS operation, only the regions to be inspected in the pictures will be retained. Refer to Figure 2 for specific explanation.

At the beginning, an industry camera captures photos in FPGA. Then, programs running on the operation system of FGPA (PS, Processing System) automatically perform CZS preprocessing on those photos according to the metadata stored in the database. Preprocessed images are then transmitted to the DPU implemented by PL, which is a special customized hardware for mapping and running the darknet-39 YOLOv3 model. With it, the process of defect inspection can be accelerated and finished. 

**Figure 8:**
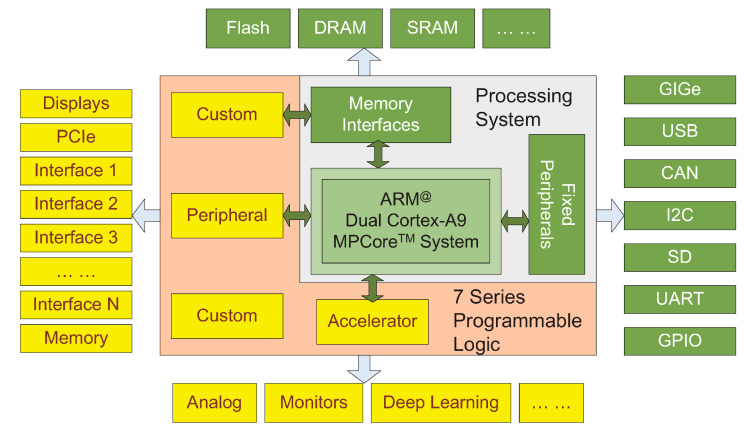
Xilinx PYNQ-Z2 architecture.

A Xilinx PYNQ-Z2 FPGA board is equipped with a ZYNQ-7020 APSoC chipset (Xilinx AMD Inc., San Jose, USA), which has both a “hard core” and a” soft core”. As shown in Figure 8, the hard core and its functions are grey and green boxes, and the soft core and its functions are orange and yellow boxes. The hard core is a 650 MHz ARM Cortex-A9 dual-core processor (Arm Inc., Cambridge, UK), running an embedded Ubuntu system (Canonical Ltd., Landon, UK). This processor supports python programming for simple processing (preprocessing image, running database, etc.) and C++ programming for calling the DPU. The soft core is the PL that can be employed by the B1152 DPU architecture in accordance with the Xilinx DNNDK 3.0 (Xilinx AMD Inc., San Jose, CA, USA) framework. Deep learning algorithms can be transformed to a format that the DPU can read and execute without fully utilizing hardware resources. The efficiency of processing 416 × 416 images of YOLOv3 is approximately 3.5 FPS, and the rated power is approximately 10 W (the power information comes from the technical documents of Xilinx PYNQ-Z2), having much better power-efficient performance than a common Central Processing Unit (CPU) or a Graphics Processing Unit (GPU) chip. This can fully meet our predesigned efficiency target. 

### 3.2. Deployment

**Figure 9:**
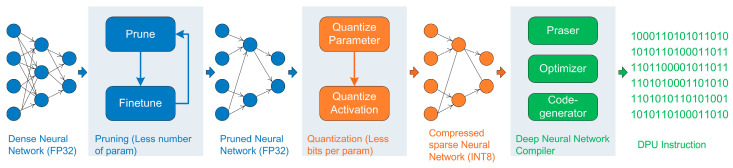
Pruning, quantifying, and compiling processes.

Deployment involves two parts, host-side deployment and FPGA-side deployment. On the FPGA side, we adopt the system version of the intelligent car hydramini, the essence of which is a customized DPU platform for PYNQ-Z2. The host side is the computer side. The host side needs to install the deep learning development platform, as well as the DNNDK 3.0. DNNDK is necessary for converting a standard deep learning model into a deployable model on PYNQ-Z2. DNNDK’s core functions include pruning, quantization and compiling. As shown in Figure 9, the blue flowchart represents the pruning operation, the orange flow chart represents quantization operation, and the green flowchart represents the compiler. 

Pruning is to obtain a new condensed network from the network pretrained by PyTorch (Meta Inc., Silicon Valley, CA, USA) or Tensorflow (Google Inc., Silicon Valley, CA, USA). Pruning consists of automatically deleting some redundant branches that do not affect the network output, and replacing variable values on network nodes with constant values of the current session. 

Quantization is to convert float-point values into fixed-point values. The first benefit from quantization is improving processing performance, using short bytes of data instead of long bytes. In general, 32-bit float-point values are replaced with 8-bit integers. As a result, the entire network is compressed. The second benefit lies in that the PYNQ-Z2′s Digital Signal Processing (DSP) units mainly support processing fixed-point values, the fixed-point values operations of which are specially optimized. 

Compiling is the deep learning algorithm being transformed into binary instruction files that can be recognized by the DPU. The functional module consists of three parts: interpreter, optimizer and code generator. The interpreter parses the quantization model and converts it into Intermediate Representation (IR). The optimizer is responsible for optimizing IR. The code generator makes optimized IR into DPU-recognized instructions. 

**Figure 10:**
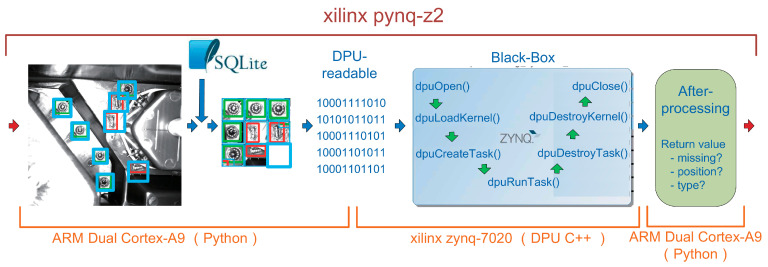
Process flow of PYNQ-Z2.

After the above three steps, a deep learning model file that is recognized by the PYNQ-Z2 DPU is generated. The file is deployed to PYNQ-Z2 and loaded on the core kernel process of the DPU. We can let the PYNQ-Z2 reload DPU Intellectual Property (IP) then use an executable program written by the DPU C++ library to invoke the deep learning model, including using API to read problem initialization parameters and analyze the output results. Deployment is finished. All above steps is shown in Figure 10. CZS and after-processing operations are undertaken by ARM (Arm Inc., Cambridge, UK), refer to Figure 2 for specific explanation. And defect inspection operations are undertaken by Xilinx ZYNQ-7020 (Xilinx AMD Inc., San Jose, CA, USA).

Access to the database and image preprocessing require a low amount of data processing resources, which are handed over to the ARM CPU for processing. In contrast, deep learning needs a lot of computing power, and FPGA is responsible for this processing work to achieve hardware acceleration. As a SoC board, PYNQ-Z2 fully realizes the seamless connection between the operating system and programmable hardware. Python and database software such as SQLite run on ARM CPU, while the DPU C++ code directly calls ZYNQ-7020 FPGA hardware resources, which optimizes the load balance of the whole process. 

## 4. Experimental Results

### 4.1. Experiment Design

**Figure 11:**
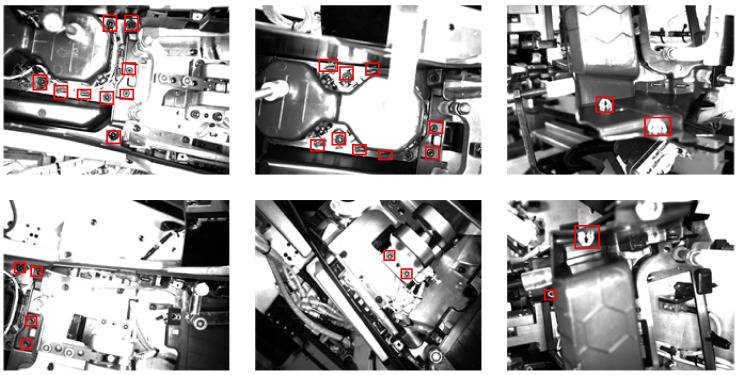
Several sample photos.

The case study gets experimental data from an automobile rubber and plastic parts manufacturer, which is a super class parts supplier for several well-known automobile brands in China, such as SAIC GM Wuling and FAW Volkswagen. Therefore, success in implementing the application system will have significance in the whole auto parts industry. With the help of industrial cameras with 10 fixed camera angles, we collected 630 photos compared with the original 63 sample photos. The photos have the same 1600 × 1200 pixels resolution and 8-bit depth. In addition, there are 16 types of defect markups. The ratio of normal and defective samples in the 630 pieces of original pictures is approximately 9:1. Several sample photos are shown in Figure 11, the red box on the pictures identify the area to be detected. The hardware of the host side mainly consists of a NVIDIA RTX3090 24 GB GPU (Nvidia Co., Silicon Valley, CA, USA), an AMD R9 3900X CPU (AMD Inc., Silicon Vallsey, CA, USA), 64 GB of DDR4 (Hynix Inc., Seoul, Korea) memory and a 4 TB mechanical hard disk (Seagate Technology, Scotts Valley, CA, USA). The software installed on the host side include Ubuntu, Compute Unified Device Architecture (CUDA) (Nvidia Co., Silicon Valley, CA, USA), Python (The Python Software Foundation, Wilmington, DE, USA), PyTorch, Tensorflow, Docker (Docker Inc., San Francisco, CA, USA) and DNNDK 3.0 (Xilinx AMD Inc., San Jose, CA, USA).

### 4.2. Host Side

On the host workstation computer side, our algorithm, the attention-based YOLOv3, is trained. The maximum number of training epochs was initially set to 300. In the training process, the training set was composed of 36139 photos after data augmentation, at a total of 11 GB. The training process took a long time, as one round of epoch took nearly 17 min. On the other hand, precision convergence was very fast. When we finished the work after 300 epochs of training, accuracy exceeded 99%.

With the pretrained model, we tested the time efficiency on the host side. The time spent is mainly divided into two parts: the loading time of the Python library is approximately 1 s, and the inspection time is approximately 0.01 s, as shown in Table 1. The loading process of the Python library is very time consuming, which can be made into a daemon, so that it is always in the loaded library state, scanning to detect changes in images, and real-time inspection.

As shown in Table 1, our algorithm’s inspection time decreased by 0.004 s, and accuracy improved by 0.2%. In sum, compared with the majority of indicators, our algorithm makes an improvement to classical YOLOv3, indicating that our algorithm achieves better performance by tailoring the backbone network.

### 4.3. FPGA Side

The efficiency and accuracy of the algorithm on the host side fully meet the industrial requirements, so the trained neural network is moved to the FPGA side. The DNNDK environment is deployed with Docker to prune, quantify and compile the trained network; and then deploy the processed neural network files to the appropriate location on PYNQ-Z2, reload the DPU kernel and compile the executable program, which completes the migration and deployment of PYNQ-Z2. Because it is necessary to use onboard Python to call the low-performance ARM CPU for database query and image preprocessing, although the performance of programmable hardware circuit is very good, the overall timeliness of the system is lower than that of the host side. As shown in Table 2, the performance of PYNQ-Z2 will be reduced to some extent, but it is also competent for missing fault inspection. The experimental results on PYNQ-Z2 also showed satisfactory performance, similar to that of the host side. A comparison of processing times of the host workstation computer and PYNQ-Z2 is shown in Table 2.

Although the process speed on PYNQ-Z2 is slower than that on the host side. The SoC cutting-edge equipment shows a reasonable efficiency with much lower power consumption than a workstation computer. Additionally, the comprehensive performance of our algorithm can meet the predesigned target of Section 1, as shown in Table 3.

### 4.4. Inference

In this study, the mean of average precision (mAP) and the intersection over union (IoU) were used as the main accuracy evaluation indices. In this case, the average recognition accuracy of 16 detection categories is calculated, and then the average value of these 16 average accuracies is the mAP. The closer the mAP is to 1, the better. Before training, the manually marked area to be detected is called the ground-truth bounding box. Later, the marked area detected by the model is called the predicted bounding box. The IoU is the intersection of these two regions divided by the union. The closer the IoU is to 1, the better, indicating that the model detection area is consistent with the manually marked area.

### 4.5. Results

**Figure 12:**
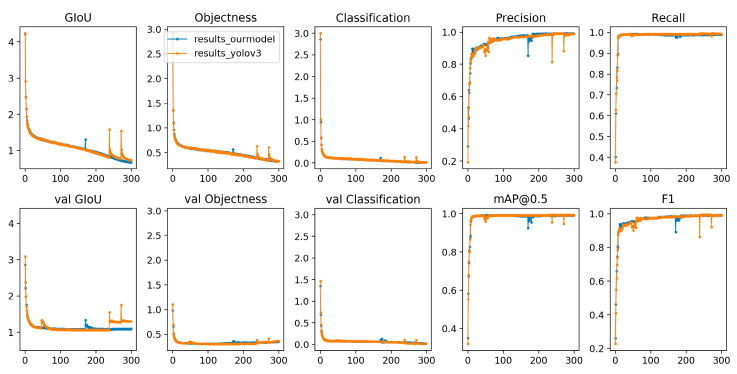
Comparison of our algorithm and classical YOLOv3.

**Figure 13:**
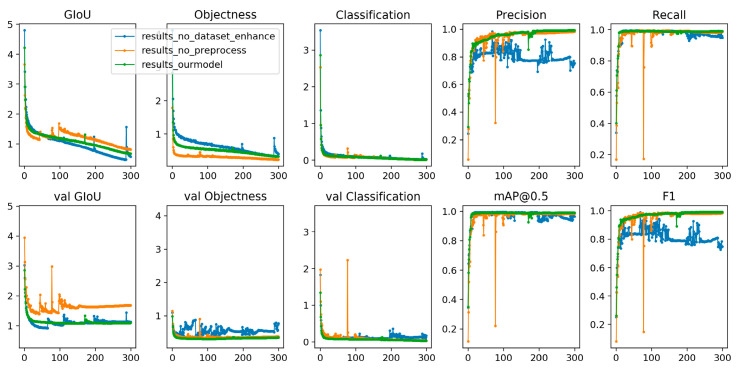
Effect of preprocessing and data augmentation on our algorithm.

This method has achieved good results on the missing faults dataset. A total of 8064 test samples are correctly classified according to type. The detection results of some examples are shown in Figure 12 and Figure 13. This method not only has good classification accuracy, but also has good positioning, timeliness and energy consumption ratio. In the past, similar defect detection research did not achieve detection accuracy over 99%, but our algorithm not only improves accuracy, but can also be promoted at an enterprise level to complete cutting-edge detection work in a fast, cheap, stable and green way. In addition to accuracy, manufacturers are concerned with the efficiency, the stability, the scalability and the comprehensive cost performance of large-scale deployment. 

In Figure 12 and Figure 13, the *x*-coordinate axis represents training times and the *y*-coordinate axis represents the value of the measurement index. 

The comparison results are shown in Table 4. For the first time in this study, FPGA is used to implement the deep learning missing installation detection system. The performance of the hardware scheme is moderate, and the price is moderate. Deployment is difficult, but the advantages are high stability and obvious design flexibility. A comparison of the performance of this algorithm with that of previous algorithms is shown in the table below.

The performance impact of an attention mechanism on the algorithm is shown in Table 5.

A comparison of the main performance indices for our detection algorithm deployed on FPGA and for similar chips is shown in Table 6.

### 4.6. Further Discussion and Analysis

At the same time, some related experiments are carried out to further verify the validity of the algorithm.

Transfer learning: When training this model, weight is trained from scratch. However, some research also states that the classical YOLOv3 algorithm can be quickly developed through transfer learning. Therefore, we consider two different training schemes: (a) training the network from scratch; (b) training the network by the transfer learning method using a classical network pretrained by the Common Objects in Context (COCO) dataset. The training time of the two methods is very similar, at approximately 48 h, and the mAP of the two methods is approximately 0.991. We think that the effect of cross-dataset transfer learning is not obvious due to the large difference between the dataset of this study and the COCO dataset.

Precision analysis: Compared with the classical YOLOv3 network, our algorithm reduces 14 neural network layers. However, after 300 rounds of training, accuracy improved from 95 to 99.2%. We think that is because the optimized version has fewer network parameters and is easier to converge than the classical version.

Error analysis: Although the recognition accuracy of this method reaches 99.2%, the premise is the existing 10 types of images. When adding a new angle image, it needs automatic recognition and automatic preprocessing. Although identification did not improve, we find that accuracy cannot reach 99.2%. Therefore, it is necessary to train a new network through transfer learning. 

FPGA side performance improvement: On the host side, the size of the input image has little impact on algorithm efficiency. However, on the FPGA side, there is a big difference in the recognition efficiency of different-sized images. The key of the problem is that image preprocessing is not based on a programmable hardware circuit, but is based on ARM soft core processing. The ARM CPU of PYNQ-Z2 has poor performance and a slow speed. If image preprocessing is also made into a programmable hardware circuit, FPGA hard core processing should be able to effectively improve the overall performance of the algorithm.

## 5. Conclusions

The traditional inspection algorithm of missing faults is limited by materials, safety, costs, etc. In this paper, a new and efficient algorithm based on an attention mechanism that can be deployed on FPGA for defects inspection is proposed. Through our algorithm, we can correctly identify the problem of missing faults in addition to achieving high precision, a fast speed and low energy consumption. The experimental results show that the accuracy of the algorithm is 99.2%, processing speed is 1.54 FPS, and energy consumption is 10 W. The algorithm can be widely deployed in the industrial field as cutting-edge equipment.

By the second quarter of 2022, there were 366, 173 and 95 articles containing YOLOv3, YOLOv4 and YOLOv5, respectively, in search titles on Web of Science. YOLOv3 is a very classical algorithm, while YOLOv4 and YOLOv5 represent an inevitable trend. In particular in the fields of robot [27] and dynamic object capture [28], YOLOv5 has made new progress, which points out the direction for future improvement in this research.

## Figures and Tables

**Table 1:** Comparison between our algorithm and YOLOv3.

	Training	Inspection	Accuracy
YOLOv3	28 FPS	0.014 s	95.0%
Our Algorithm	28 FPS	0.010 s	99.2%

**Table 2:** A comparison of processing times of workstation computer and PYNQ-Z2.

	Preprocess	1600 × 1200	416 × 416
Host	0.24 s	0.01 s	0.01 s
PYNQ-Z2	0.31 s	1.20 s	0.65 s

**Table 3:** A comparison of processing times of different algorithms on PYNQ-Z2.

	SetImage	RunTask	Deal
No preprocess, YOLOv3	0.71 s	0.45 s	1.20 s
Preprocess, YOLOv3	0.13 s	0.45 s	0.73 s
Preprocess, our algorithm	0.13 s	0.38 s	0.65 s

**Table 4:** A comparison of the performance of this algorithm with that of other representative industry defect inspection studies.

	Accuracy	1/FPS	mAP_bbox_
Nico Prappacher [23]	98%	0.153	-
Ge Liling [24]	97.2%	-	-
Ting He [25]	98.7%	0.007	-
Chunyang Xia [26]	98.4%	-	-
Junfeng Jing [16]	98%	0.046	-
Our algorithm	99.2%	0.010	0.991

**Table 5:** Performance impact of an attention mechanism.

	Accuracy	FPS	mAP_bbox_
No preprocessing	98.3%	97	0.986
No data augmentation	75.3%	97	0.948
YOLOv3	95.0%	73	0.988
Our algorithm	99.2%	97	0.991

**Table 6:** Comparison of three representative chips.

	Disadvantage/Advantage	Power Consumption	FPS
Jetson Nano	Large heat dissipationAI performance is relatively high	10 W *	4.27
Intel NCS 2	High total cost and not SoC chipIt is easy to get started	-	1.36
PYNQ-Z2	Poor performance of its CPUHas good flexibility and scalability	10 W *	1.54

* The power consumption data are based on the official instruction document of the chip.
